# Efficacy of an intermittent energy restriction diet in a primary care setting

**DOI:** 10.1007/s00394-019-02098-y

**Published:** 2019-10-11

**Authors:** R. Antoni, K. L. Johnston, C. Steele, D. Carter, M. D. Robertson, M. S. Capehorn

**Affiliations:** 1 Nutritional Sciences, Faculty of Health and Medical Sciences, University of Surrey, Guildford, UK; 2 Lighterlife UK Ltd, Cavendish House, Parkway, Harlow Business Park, Essex, UK; 3 Department of Nutritional Sciences, Faculty of Life Sciences and Medicine, Kings College London, London, UK; 4 Clifton Medical Centre, The Health Village, Rotherham Institute for Obesity, Doncaster Gate, Rotherham, UK

**Keywords:** Intermittent energy restriction, Intermittent fasting, Primary care, Weight loss, Glucose, Lipids

## Abstract

**Purpose:**

Intermittent energy restriction (IER) is a popular weight loss (WL) strategy; however, its efficacy in clinical practice remains unknown. The present study compared the effects of IER compared to continuous energy restriction (CER) on WL and cardiometabolic risk factors in primary care.

**Methods:**

A (self-selected) cohort study was conducted at the Rotherham Institute for Obesity (RIO), a primary care-based weight management service. 197(24% male) obese patients volunteered to participate and selected their diet group. IER participants (*n* = 99) consumed ~ 2600 kJ for two days/week. CER participants (*n* = 98) restricted their diet by ~ 2100 kJ/day below estimated requirements. Both interventions were delivered alongside RIO standard care. Changes in anthropometry and cardiometabolic disease risk markers (fasting biochemistry and blood pressure) were assessed after a 6-month intervention period and then participants were followed up again 6 months later (month 12).

**Results:**

27 IER patients (27%) and 39 CER patients (40%) completed the 6-month weight loss phase. Among completers, mean (SEM) WL was greater in the IER group at 6 months (5.4 ± 1.1% versus 2.8 ± 0.6%; *p* = 0.01), as were reductions in fat mass (*p* < 0.001) and improvements in systolic blood pressure (*p* < 0.001). Fasting insulin (*p* = 0.873) and diastolic blood pressure (*p* = 0.701) were reduced similarly in both groups. However, in the IER group, changes in anthropometry and blood pressure in the IER group had reverted to baseline by 12-month follow-up, whilst the CER group maintained weight loss but showed an increase in blood pressure.

**Conclusions:**

Among completers, IER resulted in superior short-term changes in anthropometry and some cardiometabolic risk factors. However, rates of attrition and weight regain were higher compared with standard care, providing important insights in the implementations of IER within a “real-life” NHS setting.

**Trial registration number:**

ISRCTN31465600.

**Electronic supplementary material:**

The online version of this article (10.1007/s00394-019-02098-y) contains supplementary material, which is available to authorized users.

## Introduction

Overweight/obesity is becoming an increasingly prevalent threat to the health and wealth of modern day societies. Related health complications include type 2 diabetes (T2DM) and cardiovascular disease which now account for a considerable proportion of annual deaths [1]. A sustained modest weight loss of 5–10% is associated with improvements in various indices of cardiometabolic health including insulin sensitivity, blood pressure and lipids [2]. The most commonly employed dietary approach to weight loss involves varying degrees of continuous energy restriction (CER). However, when confronted with an obesogenic environment favouring sedentary lifestyles and passive overeating, successful weight loss and the necessary cognitive eating restraint required for CER becomes notoriously difficult to both achieve and maintain.

In recent years, the interest surrounding intermittent energy restriction (IER) as a potential strategy to improve compliance to energy-restricted diets has increased [3] although findings from a recent systematic review comparing IER to CER demonstrate equivalence [4]. Nonetheless, studies conducted demonstrate that IER is an effective strategy for weight loss and improving cardiometabolic health markers such as fasting insulin, lipids and blood pressure when delivered in controlled settings [5], whilst weight maintenance rates have been shown to be comparable to CER [6].

A caveat to the promotion of IER within primary care is that the clinical trials to date have involved self-referring participants who are typically well motivated. No study has been conducted within an NHS obesity setting which is more reflective of the “real-life” situation and includes individuals with complex obesity. With this in mind, the present study aimed to compare the effects of IER versus CER on anthropometry, cardiometabolic health markers and long-term weight maintenance within a cohort of obese participants referred to the Rotherham Institute for Obesity (RIO). Located in South Yorkshire, RIO is a Tier 3 specialist NHS Centre offering a multi-disciplinary approach to weight management in primary care.

## Participants and methods

### Participants

Patients with obesity (BMI > 30 kg/m^2^) aged 18–65 years who had been referred to RIO by their general practitioner were recruited into the study and description of the referral criteria can be found elsewhere [7]. The study obtained a favourable opinion from the South Yorkshire NHS Ethics Committee (ref: 14-YH-0018) and was conducted in accordance with the guidelines laid down in the Declaration of Helsinki. All participants provided written informed consent. The trial was prospectively registered in ISRCTN (ISRCTN31465600).

### Study design

The present study was a parallel-armed cohort study comparing IER to CER. The weight loss intervention was 6 months, with follow-up at 1 year (i.e. 6 months following cessation of the intervention) for those participants still engaged with the practice. Participants were not randomised, but instead self-selected their dietary intervention group to promote patient autonomy and facilitate long-term successful lifestyle change. Dietary advice was provided by specialist obesity nurses.

### Dietary interventions

#### Intermittent energy restriction

On 2 days of the week, participants consumed 4 commercially available LighterLife™ very low energy formula-based Food Packs (2638 kJ: 38%, 36% and 26% of total energy as carbohydrate, protein and fat), which met daily requirements for vitamins and minerals. Participants were provided with Food Packs during the 6-month intervention phase only. On the remaining 5 days (“feed days”), participants’ food intake was self-selected, but they were asked to consume a healthy diet compliant with UK-based guidelines issued by the national institute of clinical excellence, NICE (The EatWell Plate: https://webarchive.nationalarchives.gov.uk/20120503053141/http://www.dh.gov.uk/en/Publicationsandstatistics/Publications/PublicationsPolicyAndGuidance/DH_126472). These guidelines recommend a diet based on starchy high-fibre foods; ≥ 5 portions of fruit and vegetables; lean protein sources (e.g. meat, poultry, fish and pulses); low-fat dairy (e.g. milk, cheese); and limiting intake of total fat, sugar, salt and alcohol. Advice on portion control (e.g. smaller portions, food swaps) was also provided to attain dietary targets.

#### Continuous energy restriction diet

Participants assigned to the CER diet were advised to consume a daily hypo-energetic diet of 2092 kJ (500 calories) below their estimated energy requirements, incorporating healthy eating principles (as outlined above). Advice on portion control (e.g. smaller portions, food swaps) was also provided to attain dietary targets. Requirements were calculated using the Harris Benedict equation multiplied by an appropriate physical activity factor in accordance with the standard practice at RIO [8]. All foods were self-selected by participants. The CER intervention served as the “standard treatment” control, compliant with current practice, UK NICE obesity guidelines and the 2013 NHS Commissioning Board policy criteria for Tier 3 services.

As part of the standard RIO service, all participants on both arms of the study had access to a variety of specialist facilities, resources and multidisciplinary specialists including exercise and talking therapists [7]. Participants in both groups were regularly reviewed in the clinic every month, where adherence to dietary advice (including food pack consumption) was discussed.

During the subsequent 6-month period, weight goals (continued weight loss or maintenance) were determined on an individual basis. IER participants were not specifically advised to continue ‘fasting’ (this was patient choice), whilst all participants were advised to continue following healthy eating guidance. Participants in both groups were able to still attend RIO every month for the following 6 months (unless participants in either group dropped out) and IER participants were free to continue the IER diet self-funded (if they chose to). During this time, both groups could access the other elements of RIO standard care, e.g. the gym, cooking skills classes and talking therapies.

### Study measurements

Measurements were taken before participants started their diet and then serially over the course of the study by either the specialist nurse or healthcare assistant. These measurements included weight, total body fat, fat-free mass (FFM), waist circumference, systolic and diastolic blood pressure and an overnight fasted blood sample. Body composition was assessed by bioimpedance (TANITA MC-180MA; Tanita Corp, Tokyo, Japan). Waist circumference was measured at the midpoint between the lower margin of the lowest palpable rib and top of the iliac crest at the end of normal expiration. Blood pressure was measured using an automated sphygmanometer following a 5-min rest (7670-16767; Welch Allyn, Skaneateles Falls, NY, USA).

Biochemical analyses (with the exception of insulin) were conducted at two accredited hospital laboratories (Rotherham District General Laboratory; Royal Surrey County Hospital Pathology Partnership, UK) by personnel blinded to group assignment. Fasting insulin was measured in batch upon study completion via radioimmunoassay using a commercially available kit (Merck Millipore, MA, USA; inter/intra assay CVs < 10%) by a study investigator (RA) who was blinded to group assignment at the time of the analyses.

Participants completed validated 7-day diet diaries at 3 time points: baseline (prior to commencing diet), then months 3 and 6 whilst on the diet. Diaries included pictorial guides to aid portion size estimations. All dietary analyses were carried out in diet plan 7 (Forestfield Software, Horsham, UK) using the McCance and Widdowson’s composition of foods integrated dataset. For the purpose of this study, a compliant ‘fast’ day was defined as one where energy intake was ≤ 3347 kJ, which corresponds to the very low energy threshold defined by the NICE Obesity Guidelines [9].

### Statistical analyses

Data were statistically analysed using SPSS v23 (IBM, Chicago, USA). Data were first checked for normality using the Shapiro–Wilk test, with non-normally distributed data normalised via log transformation if required to permit parametric testing. The primary analysis was a completer-only analysis, owing to the high attrition rates exhibited by both groups. The alternative approach is intention to treat (ITT) analyses which include every subject who is randomized, ignoring noncompliance, protocol deviations and withdrawal which thus maintains prognostic balance generated from the original random treatment allocation. However, in the present study complete outcome data were not available for all randomized participants, i.e. study measurements were not taken for participants who dropped out. Hence, assumptions must be made, e.g. using last observation carried forward (LOCF) or baseline observation carried forward (BOCF) which affect the validity of study outcomes, particularly in the context of the 66% attrition rate. ITT analyses for anthropometric data (for which intermediate outcomes were available) were, therefore, conducted as secondary analyses (presented in the supplementary materials).

To assess between-group differences, a repeated measures analysis of variance was used which included gender and T2DM status as covariates. Other relevant covariates (e.g. age, other comorbidities) were omitted as covariates, as initial exploratory analyses failed to find any significant interaction effects. Paired t tests were used to assess within-group changes between the various study time points. Independent-sample t tests and Chi-square test were used to compare participant characteristics at baseline. Significance was assumed when *p* < 0.05 (two tailed), and unless otherwise stated, data are expressed as mean ± standard error of the mean (SEM).

## Results

### Participant baseline characteristics and attrition

**Fig. 1:**
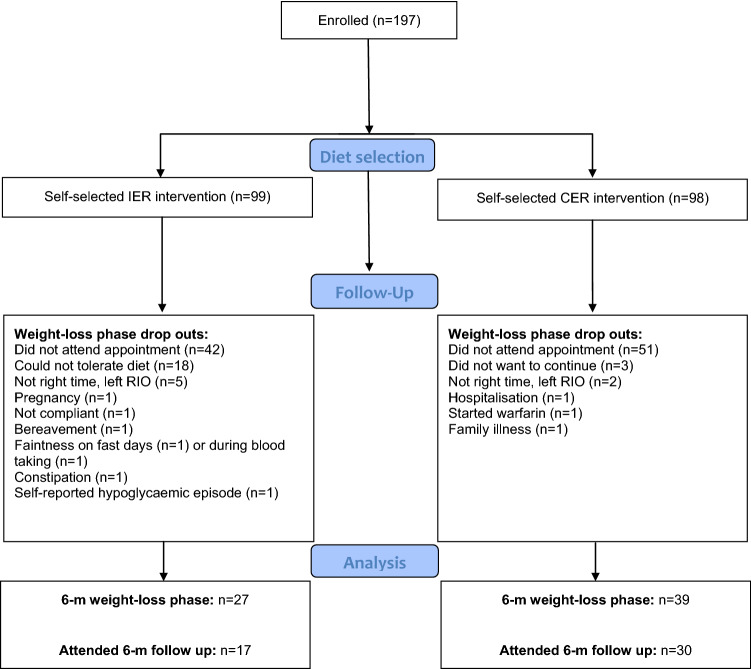
Consort diagram

Of the 197 participants (IER = 99, CER = 98) who started the study, 66 (IER = 27, CER = 39) completed the 6-month weight loss phase. Baseline characteristics of these study completers are presented in Table 1. The groups were matched for BMI, adiposity, gender and were primarily Caucasian. CER participants tended to be older (56 vs 50 years, *p* = 0.055). 131 participants withdrew from the study, with reasons for dropout detailed in the study consort diagram (Fig. 1). More dropouts occurred in the IER group (*n* = 72), of which a number of dropouts were attributed to diet-related adverse effects including faintness on fast days (*n* = 1), constipation (*n* = 1), self-reported hypoglycaemia (*n* = 1), and *n* = 18 reported that they could not tolerate the diet. Non-completers tended to have a slightly higher BMI (41.5 ± 0.7 vs. 39.6 ± 0.7 kg/m^2^; *p* = 0.070, unpaired t test); no other significant differences were noted. Of the completers of the weight loss phase, follow-up data at 1 year from study commencement were available for 47 (IER = 17, CER = 30) patients.

**Table 1:** Baseline characteristics for study completers

	IER (*n* = 27)	CER (*n* = 39)	IER vs. CER^b^
Age (years)	50 ± 2.4	56 ± 1.7	0.055
Gender (% female)	70%	74%	0.721
Ethnicity			0.402
Caucasian	*n* = 27	*n* = 38	
South Asian	*n* = 0	*n* = 1	
Comorbidities			
Type 2 diabetes	*n* = 4	*n* = 6	0.949
Cardiovascular disease	*n* = 1	*n* = 0	0.226
Hypertension	*n* = 7	*n* = 16	0.206
Sleep apnoea	*n* = 1	*n* = 3	0.504
Hypothyroidism	*n* = 2	*n* = 0	0.084
Weight (kg)	108.9 ± 3.6	111.7 ± 2.7	0.525
BMI (kg/m^2^)	39.3 ± 1.2	39.9 ± 0.8	0.675
Body fat (%)^a^			
All	40.2 ± 1.4	41.8 ± 1.0	0.322
Males	32.6 ± 2.0	36.6 ± 1.9	0.187
Females	43.4 ± 1.2	43.6 ± 0.9	0.889

^a^Bioimpedance

^b^Statistical comparisons between intermittent and continuous energy restriction groups (IER and CER, respectively) conducted via unpaired *t* tests (for continuous variables) or Chi squared (for categorical variables). Presented as mean ± SEM

### Changes over 6-month dietary intervention

#### Dietary intakes

At baseline, dietary intakes did not differ between IER and CER groups for most nutrients (Table 2), except alcohol, where intakes were higher in the IER group (*p* = 0.02, independent t test). By the end of the intervention period, both groups reported reductions in averaged weekly intakes of energy. However, this tended to be greater in the IER group (diet*time *p* = 0.076), with similar trends noted for carbohydrate (diet*time *p* = 0.029), sugars (diet*time *p* = 0.037), fat (diet*time *p* = 0.081) and salt (diet*time *p* = 0.028). Adherence to the IER protocol (i.e. two substantial ER days/week) was moderately high both 3 months into (75%) and in the final week (67%) of the 6-month weight loss phase.

**Table 2:** Dietary intakes over the course of the 6-month dietary intervention period

	IER (*n* = 18)			CER (*n* = 26)			IER vs. CER^d^
	Baseline	Month 3	Month 6	Baseline	Month 3	Month 6	
Energy intake kJ/day	6269 ± 3178	4973 ± 207^a^	4676 ± 167^b^	6773 ± 323	6242 ± 255	6168 ± 324^b^	0.076
Carbohydrate g/day	171 ± 9	122 ± 7^a^	124 ± 6^b^	180 ± 14	170 ± 8	188 ± 13	0.029
Sugars g/day	70 ± 5	71 ± 7^a^	52 ± 5^b^	71 ± 5	77 ± 8	73 ± 6	0.037
Fibre g/day	19 ± 1	16 ± 1^a^	18 ± 2^b^	18 ± 2	18 ± 2	18 ± 2	0.387
Fat g/day	53 ± 5	44 ± 3^c^	36 ± 2^bc^	55 ± 3	55 ± 4	49 ± 3	0.081
Saturated fat g/day	18 ± 2	20 ± 1^c^	19 ± 2^bc^	20 ± 1	20 ± 2	18 ± 1	0.333
Protein g/day	71 ± 4	66 ± 3	63 ± 2^b^	79 ± 5	71 ± 3^a^	69 ± 4^b^	0.797
Alcohol g/day	112 ± 40	80 ± 23^a^	81 ± 30	13 ± 6	11 ± 6	45 ± 32	0.310
Salt g/day	6.4 ± 0.3	5.6 ± 0.4^a^	6.1 ± 0.7	6.2 ± 0.3	5.6 ± 0.3	6.1 ± 0.5	0.028

Data are presented as mean ± SEM

^a,b^Significant within-group difference between ^a^ baseline and week 12; ^b^baseline and week 24; ^c^week 12 and 24 (paired *t* test)

^d^*p* values represent the interaction between study group and assessment period (2-way repeated measures ANOVA)

### Changes in body composition and circumferences

**Fig. 2:**
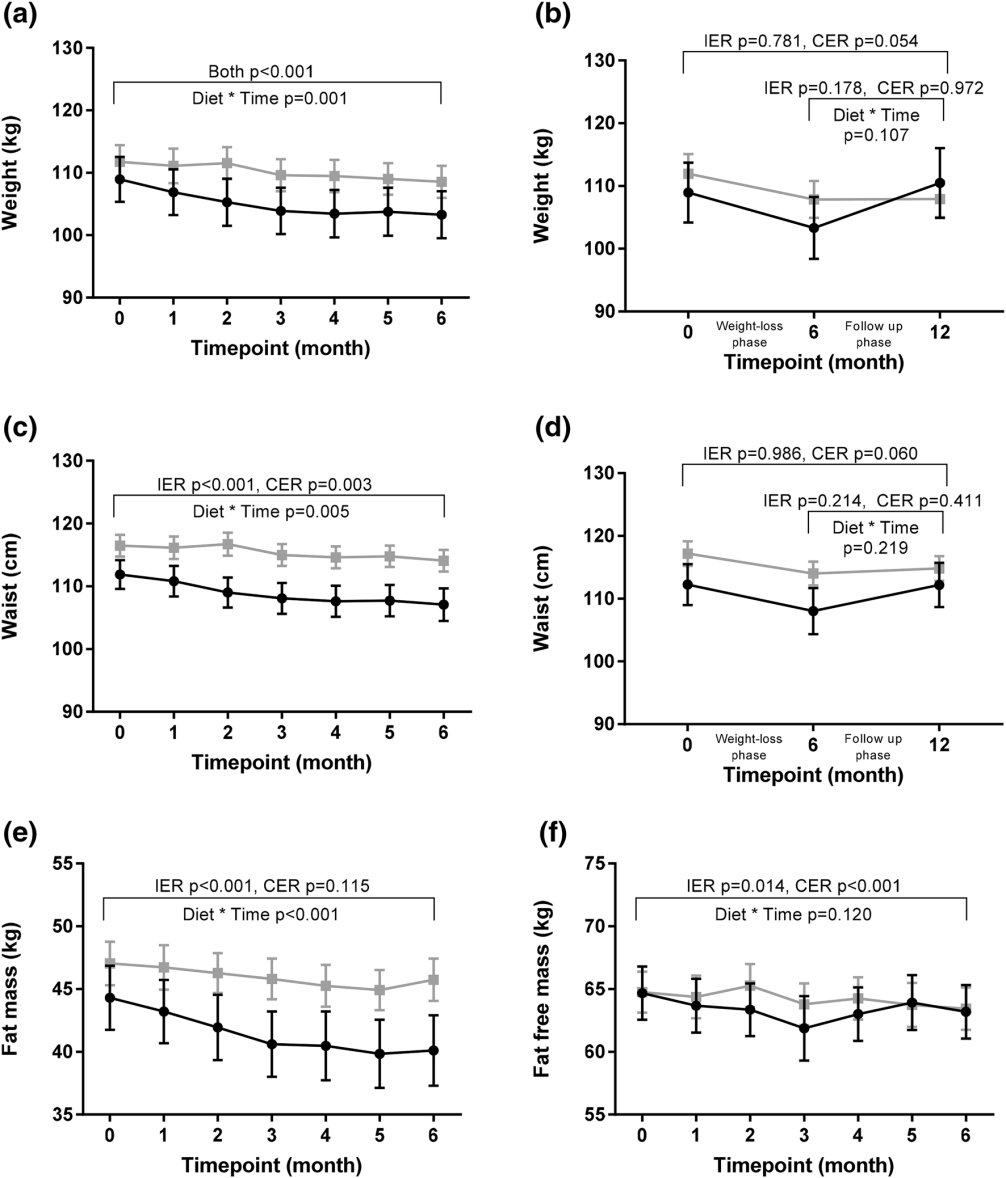
Changes in anthropometry. Intermittent energy restriction (IER): black circle. Continuous energy restriction (CER): grey square. Presented as mean ± SEM. Statistical comparisons between groups conducted via repeated measures analysis of variance. Within-group changes assessed via paired *t* tests. *N* = 27 IER, *N* = 39 CER (**a**, **c**, **e**, **f**) or *N* = 17 IER, *n* = 30 CER (**b**, **d**)

As depicted in Fig. 2, among completers mean weight loss was significantly greater in the IER group (5.4 ± 1.1%) versus the CER group (2.8 ± 0.6%) at 6 months (diet*time *p* = 0.001), with a mean difference of − 1.8 kg [95% CI − 3.2 to − 0.4 kg]. There was a significantly greater reduction in fat mass in the IER group (mean difference − 2.7 kg [95% CI − 3.5 to − 1.8 kg]; diet*time *p* < 0.001), as well as in waist circumference (mean difference − 1.6 cm [95% CI − 2.9 to − 0.3 cm]; diet*time *p* = 0.005). FFM was comparably reduced in both groups (diet*time *p* = 0.120).

Statistical outcomes for LOCF and BOCF ITT statistical analyses on anthropometric data are presented in Supplementary Table 1. Both ITT statistical approaches found that reductions in FM tended to be greater (diet*time *p* ≤ 0.081) in the IER group versus the CER group. Changes in body weight (IER − 2.0% ± 0.4% and CER − 1.6 ± 0.3%; *p* ≥ 0.264), waist circumference (*p* ≥ 0.253) and FFM (*p* ≥ 0.460) were statistically comparable between the dietary groups.

### Changes in cardiometabolic risk factors

Changes in cardiometabolic risk factors are presented in Table 3. With regard to glycaemic control indices, there were no significant between-group differences in the changes in fasting glucose (diet*time *p* = 0.143) or insulin (diet*time *p* = 0.873). Neither intervention resulted in significant changes in total cholesterol (TOTC) or LDL cholesterol (diet*time *p* ≥ 0.349), whereas HDL cholesterol (*p* = 0.007 paired *t* test) and triacylglycerol (*p* = 0.008 paired t test) increased in the IER group only although neither achieved between-group significance (diet*time both *p* ≥ 0.127). Next, for systolic blood pressure, the relative reduction was greater in the IER group (diet*time *p* < 0.001) with a mean difference of − 8.6 mmHg [95% CI − 12.7 to − 4.4 mHg], whereas changes in diastolic blood pressure were comparable (diet*time *p* = 0.701). Lastly, high sensitivity CRP (an inflammatory marker) did not change significantly in either group (diet*time *p* = 0.873).

**Table 3:** Changes in cardiometabolic risk factors over the course of the 6-month dietary intervention period

	IER (*n* = 27)			CER (*n* = 39)			IER vs. CER^b^
	Baseline	Month 3	Month 6	Baseline	Month 3	Month 6	
Fasting glucose (mmol/l)	5.1 ± 0.2	5.1 ± 0.1	4.9 ± 0.1	5.3 ± 0.3	5.2 ± 0.2	5.3 ± 0.2	0.144
Fasting insulin (uU/ml)	24.2 ± 2.2	21.1 ± 1.8	19.1 ± 2.2^a^	28.6 ± 3.6	26.3 ± 3.0	25.3 ± 2.0	0.873
Total cholesterol (mmol/l)	4.7 ± 0.2	4.2 ± 0.2	4.6 ± 0.1	4.7 ± 0.2	4.6 ± 0.2	4.6 ± 0.2	0.376
LDL cholesterol (mmol/l)	2.9 ± 0.1	2.7 ± 0.1	2.9 ± 0.1	2.9 ± 0.1	2.8 ± 0.1	2.8 ± 0.1	0.349
HDL cholesterol (mmol/l)	1.17 ± 0.01	1.17 ± 0.05	1.25 ± 0.04^a^	1.16 ± 0.04	1.17 ± 0.04	1.18 ± 0.04	0.127
Triacylglycerol (mmol/l)	1.4 ± 0.1	1.2 ± 0.1	1.2 ± 0.1^a^	1.4 ± 0.1	1.3 ± 0.1	1.4 ± 0.1	0.326
High sensitivity CRP (mg/L)	7.6 ± 2.5	7.3 ± 2.4	4.9 ± 1.2	5.9 ± 1.0	8.2 ± 1.5	5.3 ± 0.8	0.873
Systolic blood pressure (mmHg)	129 ± 2	125 ± 2	122 ± 2^a^	116 ± 2	124 ± 2	123 ± 2^a^	< 0.001
Diastolic blood pressure (mmHg)	77 ± 2	74 ± 2	72 ± 1^a^	74 ± 1	72 ± 2	71 ± 1^a^	0.701

Data are presented as mean ± SEM

^a^Significant within-group difference between baseline and month 6 (paired *t* test)

^b^*p* values represent the interaction between study group and assessment period (2-way repeated measures ANOVA)

### 6-month post-intervention follow-up

1 year after the start of the intervention, there were 17 (17%) and 30 (31%) patients still engaged with RIO who had completed the IER and CER interventions, respectively.

At one-year follow-up (Fig. 2), anthropometric measures (weight, waist circumference) had returned back to baseline levels among IER participants (*p* ≥ 0.781 baseline vs 1 year, paired *t* test). This contrasted with CER participants, who tended to maintain the reductions in these anthropometric measures (*p* ≤ 0.054 baseline vs 1 year, paired *t* test).

Some clinical data, including blood pressure, were available at the 1 year follow-up. Relative to baseline, for both groups, blood pressure parameters returned close to baseline values (*p* > 0.05, paired *t* test), whilst systolic blood pressure was greater at 1 year in the CER group versus baseline (*p* = 0.015 paired *t* test).

#### Retrospective power calculations

For change in body weight, retrospective power calculations determined that at a two-sided 0.05 significance level, the study had 45% power to detect a mean difference of 2.5 kg between treatment groups (IER vs. CER), based on a standard deviation of 5.4 kg. To attain 80% power utilising an analogous parallel-armed study design, 150 participants would be required per group.

## Discussion

### Summary

This study aimed to compare the effects of IER versus CER on anthropometry and cardiometabolic risk factors within a primary care based Tier 3 NHS weight management service. Importantly, the study also followed up those patients who were still actively engaged with RIO six months after the end of the intervention period to explore whether either diet was able to provide any long-lasting benefits. The study found greater degrees of weight loss with IER, but also greater attrition and weight regain following a 6-month follow-up period. It should be noted that the high attrition rates and subsequent underpowering of the study represent significant limitations of the study. Nonetheless, findings from the present study provide useful insights into the implementation of IER within a “real-life” NHS setting and highlight several areas worthy of further investigation. Key findings shall be discussed in turn.

Both CER and IER exhibited a significant weight loss of 3–5%, respectively, surpassing weight loss thresholds deemed to be clinically significant by NICE [9]. Indeed, both groups exhibited an improvement in at least one cardiometabolic health parameter. The IER group experienced a greater reduction in weight and associated adiposity after the 6-month intervention and exhibited superior improvements in some cardiometabolic health parameters including reductions in systolic blood pressure and in fasting triacylglycerol.

However, for those patients who were still engaged with RIO 1 year after the beginning of the intervention, those in the IER group also displayed the greatest degree of weight regain following cessation of the initial 6-month diet phase. Moreover, the IER intervention exhibited higher rates of attrition (73% versus 61%). Of these drop outs, 28% of IER participants either could not tolerate the diet or dropped out due to faintness or self-reported hypoglycaemia (*n* = 1) on fast days. It should be noted that patients were not randomised to the dietary intervention, but self-selected to mimic what happens in day- to-day practice and promote patient autonomy.

Initial weight trends are consistent with self-reported dietary intake data, which showed that IER facilitated a greater overall reduction in energy intake. Unfortunately, dietary intakes were not assessed at 1 year follow-up, but presumably IER participants resumed normal eating patterns which might explain this weight regain. Although both groups were provided with healthy eating advice and supported, more research is required to assess how to optimise IER diets and dietary advice to promote improved tolerance and weight maintenance within such setting.

### Comparison with existing data

These data are not in agreement with the popular speculation that IER might prove easier to follow as individuals need not energy restrict every day. Our data also contrast with a recent study of alternate day fasting which showed no differences in weight regain following 6 months of follow-up [6]. A key difference is that the current study involved patients with complex obesity who were referred to a specialist obesity service within the NHS. Moreover, it is also worth noting that most patients attending RIO are those patients who have previous struggled to lose weight or failed on more traditional Tier 2 weight loss interventions.

The blood lipid data are in keeping with research demonstrating that IER can positively modulate triacylglycerol metabolism [5]. With regard to systolic blood pressure, the majority of prior research shows comparable changes with CER, and so it is interesting that the present study and another utilising near identical dietary protocols [5] have shown IER to exert a greater reducing effect. However, much like weight trends, these improvements in systolic blood pressure proved to be transient, depreciating over the course of the follow-up period.

### Strengths and limitations

A particular strength of the study is that it was conducted in clinical practice and thus fills a vital knowledge gap in the IER research field. In addition, the 1 year follow-up period for those patients still engaged with the weight loss service provides some insight into the longer-term sustainability of IER.

The study does have some limitations which are important to note. Firstly, the high (66%) attrition rates within this cohort with complex obesity limit the external validity of the study. Moreover, by limiting the available sample size for the study, the statistical power of the study was also reduced which increased the risk of type 2 error (false negative). It should be noted, however, that attrition rates within the CER group, which reflects engagement with standard care, are on a par with published attrition rates for RIO and other Tier 3 specialist obesity services [10] and these findings ultimately represent “real world” data. Attrition rates, combined with a lack of follow-up data for participants who did not complete the dietary interventions, also limited the ITT analyses (included as supplementary materials) due to the assumptions required when accounting for missing observations. Thus, a completer-only analysis was used as the primary statistical method.

Next, personnel conducting the anthropometric assessments (through interaction with participants) were unblinded to diet assignment. Additionally, a lack of details on confounding factors such as medication, smoking and physical activity mean results cannot directly prove the association between the dietary interventions and study outcomes were causal. Furthermore, with a higher proportion of females in a predominantly Caucasian population, applicability of these results to the wider population is limited. Lastly, is the use of bioimpedance which sensitive to hydration status and hormonal status. Moreover, ensuring standard conditions are met before anthropometric measurements, such as prior physical activity, dietary intake and using the same observer are essential for accuracy and reproducibility, however were not always practical given the nature and location of the study.

### Implications for research and clinical practice

Overall, results from the present study comparing the long-term effects of IER with CER within an NHS weight management setting show that IER may be more effective at reducing body weight and improving some cardiometabolic risk factors in the short term. However, it was also associated with higher rates of attrition and weight regain following cessation of the 6-month weight loss phase when compared with standard care in this cohort of patients with complex obesity. Considerations for future research include a multiple-centre trial to assess the effectiveness of IER in the wider population, including its efficacy across various ages and ethnic groups. But first, future research is required to establish factors affecting short- and long-term acceptability of IER, and how best to optimise dietary advice to facilitate long-term compliance and weight maintenance.

## Electronic supplementary material

Below is the link to the electronic supplementary material.

Supplementary material 1 (DOCX 14 kb)
