# Supplementary material for: Efficacy of an intermittent energy restriction diet in a primary care setting
Source: Eur J Nutr. 2019 Oct 11;59(6):2805–12. doi: 10.1007/s00394-019-02098-y (PMC7413919; doi:10.1007/s00394-019-02098-y)
Supplement: Supplementary file 1 — Supplementary material 1 (DOCX 14 kb) [file 394_2019_2098_MOESM1_ESM.docx]

**Supplementary Table 1 - Changes in anthropometry over 6 month dietary intervention phase for all participants (completers and non-completers) using two intention to treat statistical approaches – last observation carried forward (LOCF) and baseline observation carried forward (BOCF)**

|  | **LOCF** | | | | **BOCF** | | | |
| --- | --- | --- | --- | --- | --- | --- | --- | --- |
| **Outcome** | **Baseline** | **Month 3** | **Month 6** | **P value^1^** | **Baseline** | **Month 3** | **Month 6** | **P value^1^** |
| **Weight** | | | | | | | | |
| *IER* | 113.9±2.5 | 111.8±2.5 | 111.7±2.6 ^a^ | 0.264 | 113.9±2.5 | 111.7±2.5 | 112.1±2.6 ^a^ | 0.328 |
| *CER* | 113±2.1 | 111.9±2.0 | 111.4±2.0 ^a^ |  | 113±2.1 | 112.0±2.0 | 112.0±2.1 |  |
| **Waist (cm)** | | | | | | | | |
| *IER* | 116.3±1.5 | 113.8±1.7 | 114.3±1.6 ^a^ | 0.253 | 116.3±1.5 | 114.3±1.8 | 115.0±1.6 ^a^ | 0.304 |
| *CER* | 118.0±1.4 | 116.9±1.3 | 116.7±1.3 |  | 118.0±1.4 | 117.2±1.3 | 117.1±1.4 |  |
| **Fat mass (kg)** | | | | | | | | |
| *IER* | 49.3±1.7 | 47.6±1.7 | 47.4±1.7 ^a^ | 0.034^2^ | 49.3±1.7 | 47.8±1.7 | 48.1±1.7 ^a^ | 0.081^3^ |
| *CER* | 48.5±1.3 | 47.7±1.3 | 47.6±1.3 ^a^ |  | 48.5±1.3 | 47.5±1.3 | 48.0±1.3 |  |
| **Fat free mass (kg)** | | | | | | | | |
| *IER* | 64.3±1.2 | 63.5±1.3 | 63.9±1.2 | 0.484 | 64.3±1.2 | 63.4±1.3 | 63.9±1.2 ^a^ | 0.460 |
| *CER* | 64.7±1.2 | 64.1±1.2 | 64.0±1.2 |  | 64.7±1.2 | 64.2±1.2 | 64.2±1.2 ^a^ |  |

N=99 (IER) and n=98 (CER)

Data presented as means and their standard errors

^a^ Statistically significant between baseline and month 6 (paired t test) p<0.05

^1^ Diet*time interaction (repeated measures ANOVA)

^2^ Mean difference -1.2 kg [95% CIs -1.6 to -0.8 kg]

^3^ Mean difference -0.5 kg [95% CIs -1.1 to -0.1 kg]
